# Volume Matters: Improved Outcomes for Patients Presenting to High-Volume Emergency Departments with Atrial Flutter and Fibrillation

**DOI:** 10.1371/journal.pone.0165894

**Published:** 2016-11-04

**Authors:** Rhonda J. Rosychuk, Michelle M. Graham, Brian R. Holroyd, Brian H. Rowe

**Affiliations:** 1 Department of Pediatrics, University of Alberta, Edmonton, Alberta, Canada; 2 Women & Children’s Health Research Institute, Edmonton, Alberta, Canada; 3 Department of Medicine, University of Alberta, Edmonton, Alberta, Canada; 4 Department of Emergency Medicine, University of Alberta, Edmonton, Alberta, Canada; 5 Alberta Health Services, Edmonton, Alberta, Canada; 6 School of Public Health, University of Alberta, Edmonton, Alberta, Canada; Yokohama City University, JAPAN

## Abstract

**Objectives:**

Clinical familiarity plays a role in health outcomes; the relationship between emergency department (ED) volume and outcomes for atrial fibrillation and flutter (AFF) are not clear. We compared ED presentation outcomes for AFF between high (HV) and low volume (LV) EDs in Alberta, Canada.

**Methods:**

45,372 AFF presentations for patients aged ≥ 35 years from all 104 EDs in Alberta during 1999 to 2011 using administrative health databases formed a retrospective cohort. EDs were grouped by annual AFF volume: 11 high (>100 presentations) or 93 low (≤100 presentations). Outcomes included hospital admission rate, return to ED for AFF within 30 and 90 days, and death within 30 and 90 days. Analyses included statistical tests and mixed effects modeling.

**Results:**

Mean age at ED presentation was 69.8 years (52% male). HV ED presentations were associated with lower admissions (adjusted odds ratio [aOR] = 0.68, 95% confidence interval [CI] 0.64, 0.72; p-value [p]<0.001), ED returns at 90 (aOR = 0.81, 95% CI 0.73, 0.90; p<0.001) days, and a higher likelihood of specialist visits at 30 (aOR = 1.81, 95% CI 1.68, 1.94; p<0.001) and 90 (aOR = 1.82, 95% CI 1.76, 2.03; p<0.001) days. For admitted patients, there were fewer returns to HV EDs at 30 (aOR = 0.37, 95% CI 0.15, 0.87; p = 0.02) and 90 (aOR = 0.48, 95% CI 0.26, 0.89; p = 0.02) days after hospital discharge. There was no difference in death between the two groups.

**Conclusions:**

AFF patients presenting to HV EDs experienced fewer admissions and AFF ED revisit and higher specialist referrals compared to LV EDs.

## Introduction

Atrial fibrillation and flutter (AFF) are the most common arrhythmias seen in the outpatient setting and affect more than 300,000 adult Canadians.[1] Due to their acuity and patient concerns, emergency department (ED) presentations are common, and despite the existence of clinical practice guidelines for AFF,[2,3] treatment in the ED varies.[4] For example; rate control practices, decisions regarding prompt cardioversion (chemical vs electrical), specialist referrals and follow-up time vary according to age, sex, socio-economic status, location and hospital setting (e.g., rural vs urban). ED management and follow-up vary, and outcomes could be improved.

One factor that has been explored elsewhere in health outcomes research is the role played by clinician familiarity with the condition. For example, sites with higher volume cases often perform better than sites with less familiarity and lower caseloads.[5,6] In the ED, higher volumes have been associated with lower in-hospital death in multiple conditions (e.g., pneumonia, heart failure, sepsis, stroke, respiratory failure, gastrointestinal haemorrhage, renal failure).[7] These conditions have high associated acute and sub-acute mortality compared to AFF, which is associated with long-term comorbidity (e.g., stroke, heart failure) and longer-term mortality. To our knowledge, the relationships between ED volume and outcomes for AFF have not been explored. We compare several outcomes from an ED presentation for AFF, including return ED presentation, follow-up and death (all within 30 and 90 days of ED presentation for AFF), in high and low volume EDs in one large Canadian province.

## Materials and Methods

### Study Design

This cohort study used data from the Ambulatory Care Classification System (ACCS) database in the province of Alberta, Canada during April 1, 1999, to March 31, 2011. The study was approved by the Health Research Ethics Board of the University of Alberta (#Pro00025785).

### Study Population and Data Sources

The ACCS provincial database was developed by Alberta Health to track ambulatory care visits, such as ED presentations, within government-funded facilities in the Alberta.[8] Subsequently, the National Ambulatory Care Reporting System (NACRS) was created and ACCS data now populates this national database.[9] All Albertans access health care at no direct personal cost in a uniform single-payer health system. All ED encounters in >100 EDs are entered into computerized abstracts that constitute the majority of records (there are some ambulatory care clinical sites included). Using a uniform protocol, trained and supervised medical records nosologists code each chart using ICD-9-CM[10](before April 1, 2002) or ICD-10-CA[11] (April 1, 2002 onward) diagnostic codes. Each record represents a unique service and contains visit start and end dates and times, diagnoses, and disposition status. While ACCS includes data on every patient service occurring in an Alberta ED, the data for non-Albertans could not be linked to other data sources.

Demographic data were obtained by linkage with the annual Alberta Health Care Insurance Plan cumulative registry file and physician visits to non-ED settings after ED discharge were obtained from the Physician Claim File. Alberta Vital Statistics provided data on deaths 90 days after the ED presentation and the Discharged Abstract Database provided start and end dates and times of hospitalizations.

Diagnostic information in ACCS consisted of a main ambulatory diagnosis field, and up to 9 additional fields. To be considered an ED presentation for AFF, the main (first) diagnosis field had to have diagnostic codes 427.3x “Atrial Fibrillation and Flutter” (includes 427.31 and 427.32) or I48.x “Atrial Fibrillation and Flutter” (includes I48.0 and I48.1). This database is both reliable and valid. ED presentations were extracted for Alberta residents who matched the case definition and whose age at the ED presentation was ≥35 years.

Demographic variables extracted were age in years at ED presentation, sex (male or female), and a proxy for socio-economic/cultural status (e.g., based on age and subsidy groups). The ED data included the start and end dates and time for the ED presentation. Triage level (the Canadian Triage and Acuity Scale[12] [CTAS]) represents the urgency of the ED presentation and became mandatory for all EDs in April 1, 2006: resuscitation (CTAS 1), emergency (CTAS 2), urgent (CTAS 3), semi-urgent (CTAS 4), and non-urgent (CTAS 5). Each ED presentation had 10 fields for interventions coded according to the Canadian Classification of Interventions (CCI).[13] The 10 fields were used to identify electrical cardioversion (1.HZ.09 “Stimulation, heart NEC”). All patients departing an ED are given a standardized disposition classification (e.g., left against medical advice, transferred) according to the manner in which they are released; however, most cases fall into one of two categories (i.e., discharged, admitted).

Physician visits to non-ED settings (follow-up visits hereafter) within 365 days of the ED presentation were extracted. Follow-up visit data included the date of the visit and specialty of physician. Physician visits to non-ED settings were also extracted for the 365 days prior to the ED visit and these prior visits were also used to determine comorbidities according to a standard coding scheme[14] and to calculate the Charlson Comorbidity Index based on the Deyo ICD-9 approach.[15] For this study, a specialist was defined as cardiology or internal medicine, as these require a referral from a health care professional to provide patient assessment.

An ED was classified as high volume (HV) or low volume (LV) if the average number of AFF presentations per year was >100 or ≤100, respectively (i.e., the number of presentations over all study years divided by 12 had to exceed 100 to be considered a HV ED). All patient presentations for AFF (≥1 presentation per patient) to the ED were used in this calculation. While this number is not validated, it distinguished the LV rural hospitals from urban and regional hospitals EDs in this sample and represented a natural breakpoint of the volume per year (i.e., no EDs had values between 88 and 146 presentations per year). While the EDs may also vary on other characteristics, such characteristics were not provided because of privacy regulations.

### Outcome Measures

Outcomes of interest included hospital admission at index ED presentation, return ED presentation for AFF within 30 and 90 days, and death within 30 and 90 days of ED presentation. For ED presentations that resulted in discharge, a specialist visit within 30 and 90 days was also of interest.

### Analysis

One ED presentation for AFF per patient was randomly selected per fiscal year and these index ED presentations were analyzed. Counts, percents, means, standard deviations (SDs), medians and interquartile ranges (IQRs) summarize data. For most ED presentations and outcomes, the 30 and 90 day values were calculated on the date and time of the start of the index ED presentation. For the ED presentations that ended with hospitalization, 30 and 90 days from the end of hospitalization were used for the return ED presentation for AFF outcomes. Mixed-effects logistic regression models were developed with volume category as the primary predictor of interest and with a random effect (intercept) for ED facility. A patient random effect model for patients was not developed (to account for the correlation of potentially multiple index ED presentations for a patient during different fiscal years) because a relatively small number of patients presented more than once and multiple presenters would not influence results substantively. The large data set meant including both types of random effects in a non-nested model was not feasible. In addition, multivariable models with a set of other variables were fit along with the predictor of interest. All variables were included in these models and no variables were removed. Unadjusted odds ratios (OR) and adjusted ORs (aORs) with 95% confidence intervals (CIs) are provided for the outcomes. A p-value (p) less than 0.05 was considered to be statistically significant. The c-index was determined for each multivariable model and residual diagnostic plots were examined for model fit. The c-index was greater than 0.75 for each model and the residual diagnostics did not indicate lack of fit. Data were analyzed using TIBCO Spotfire S+ (Version 8.1.1 for Linux, TIBCO Software Inc., Palo Alto, CA. 2008) and SAS (Version 9.2, SAS Institute Inc., Cary, NC. 2010). The SAS GLIMMIX procedure with a Laplace estimation option was used for the mixed models.

## Results

### Characteristics of EDs

There were 104 designated EDs in Alberta during the study period. Of these, 11 were considered HV for AFF. Most (8/11) HV EDs were located in the most urban areas of the province, the greater metropolitan areas of Edmonton and Calgary. HV EDs had a median of 257.8 presentations for AFF per ED per year (min = 147.7, max = 503.8) and LV EDs had a median of 18.8 (min = 0.1, max = 87.6). Overall, AFF patients in HV EDs stayed longer than those seen in LV EDs (5.3 vs. 2.1 hours; p < 0.001).

### Characteristics of Study Subjects

During the study period, 63,398 ED presentations were extracted (32,104 unique patients) with a primary diagnosis of AFF. With the random selection of one AFF presentation per patient per fiscal year, 45,372 ED presentations were analysed. Most patients (24,136, 75%) had one presentation (295 [1%] had >5). The majority of presentations were made by males (23,366, 51.5%), non-Aboriginal seniors (30,248, 66.7%), and those living in urban areas (34,733, 76.6%). The average age was 69.8 years (SD = 13.4). The median Charlson Comorbidity Index was 1 (IQR = 0, 2) and prior history of hypertension (20,020, 44.1%) and cardiac arrhythmias (17,127, 37.8%) were the most common comorbidities identified in the year prior to the ED presentation.

The median numbers of physician and specialist visits in the year prior to the ED presentation for AFF were 19 (IQR = 10, 34) and 1 (IQR = 0, 4), respectively. Approximately 20% (9,290) of presentations had at least one ED presentation for AFF in the previous year (based on 42,290 index presentations [93.2%] with a full year of prior ED presentations for AFF extracted). The majority of presentations occurred during weekdays (Monday-Friday, 34,252, 75.5%) and during 8:00am to 3:59pm (22,897, 50.5%).

Most of the patient characteristics were similar by volume (Table 1). HV EDs had higher proportions of urban residents (94.6%) and lower proportions of prior cardiac arrhythmias (35.1%) than LV EDs. HV EDs had longer lengths of stays, more cardioversions performed, and fewer hospital admissions than LV EDs (Table 1 and Table in S1 Table).

**Table 1:** Characteristics.

	Low AFF Volume	High AFF Volume
	(≤ 100 AFF ED presentations/year)	(> 100 AFF ED presentations/year)
EDs, n	93	11
ED presentations for AFF, n	17,596	27,776
ED presentations for AFF perED per year, median (IQR)	18.8 (10.4, 30.9)	257.8 (215.4, 320.0)
Female, n (%)	8,241 (46.8)	13,765 (49.6)
Age, mean (SD)	70.3 (13.2)	69.6 (13.5)
Socio-economic Proxy, n (%)		
Aged < 65 years		
First Nations	351 (2.0)	189 (0.7)
Government Sponsored Programs	809 (4.6)	1,019 (3.7)
Human Services Recipient	267 (1.5)	560 (2.0)
Other	4,038 (22.9)	7,540 (27.1)
Aged ≥65 years		
First Nations	265 (1.5)	86 (0.3)
Non-First Nations	11,866 (67.4)	18,382 (66.2)
Residence, n (%) (3 missing)		
Rural	9,143 (52.0)	1,496 (5.4)
Urban	8,453 (48.0)	26,277 (94.6)
Charlson Comorbidity Index, median (IQR)	1 (0, 2)	1 (0, 2)
Comorbidities, n (%)		
Diabetes	529 (3.0)	878 (3.2)
Depression	1,510(8.6)	3,174 (11.4)
Hypertension	7,993 (45.4)	12,027 (43.3)
Dementia	432 (2.5)	762 (2.7)
Anemia	270 (1.5)	526 (1.9)
Renal failure	401 (2.3)	880 (3.2)
Cancer	1,069(6.1)	1,930 (6.9)
Chronic pulmonary disease	2,700(15.3)	3,869 (13.9)
Myocardial infarction	827 (4.7)	1,508 (5.4)
Heart failure	3,076 (17.5)	4,116 (14.8)
Cardiac arrhythmias	7,376 (41.9)	9,751(35.1)
Triage Level, n (%)		
Resuscitation (1)	76 (0.4)	145 (0.5)
Emergency (2)	2,005 (11.4)	11,561 (41.6)
Urgent (3)	5,135 (29.2)	8,940 (32.2)
Semi-Urgent (4)	2,384 (13.5)	1,040 (3.7)
Non-Urgent (5)	1,614 (9.2)	124 (0.4)
Missing	6,382 (36.3)	5,966 (21.5)
Cardioversion, n (%)	435 (2.5)	3,922 (14.1)
Length of Stay in hours, n	16,124	26,262
median (IQR)	2.1 (1.1, 4.0)	5.3 (3.4, 8.5)
At least 1 ED presentation for AFF in previous 365 days, n (%)†	3,898 (22.2)	5,392 (19.4)
ED presentations for AFF in previous 365 days for those with previous ED presentations, median (IQR) †	1 (1, 2)	1 (1, 2)
Hospitalized at index ED presentation for AFF, n (%)	6,777 (38.5)	8,729 (31.4)
Physician office visits in previous 365 days, median (IQR)	18 (9, 31)	20 (10, 35)
Specialist visits in previous 365 days, median (IQR)	0 (0, 3)	1 (0, 6)

† 3,082 index ED presentations for AFF were prior to April 1, 2000, and thus did not have the full 365 days prior of observation.

#### Outcomes

AFF presentations were less likely to result in admission in HV EDs (OR = 0.72, 95%CI 0.69, 0.75; p<0.001), even when adjusted by covariates (aOR = 0.68, 95%CI 0.64, 0.72; p<0.001) (Table 2). Admitted patients at HV EDs were less likely to return to an ED for AFF at 30 (aOR = 0.37, 95%CI 0.15, 0.87; p = 0.02) and 90 (aOR = 0.48, 95%CI 0.26, 0.89; p = 0.02) days after discharge from the hospital than admitted patients at LV EDs.

**Table 2:** Outcomes.

Outcomes	n (%)	Unadjusted OR (95% CI) [p-value]	Adjusted OR* (95% CI) [p-value]
All Presentations (n = 45,372)			
Admission	15,506 (34.2)		
High volume	8,729 (31.4)	0.72 (0.69, 0.75) [<0.001]	0.68(0.64, 0.72) [<0.001]
Low volume	6,777 (38.5)	Reference	Reference
Death within 30 days	1,114 (2.5)		
High volume	761 (2.7)	1.38 (0.72, 2.44) [0.36]	0.58 (0.27, 1.23) [0.16]
Low volume	353 (2.0)	Reference	Reference
Death within 90 days	2,098 (4.6)		
High volume	1,390 (5.0)	1.23 (0.80, 1.88) [0.35]	0.55 (0.49, 1.48) [0.57]
Low volume	708 (4.0)	Reference	Reference
Discharged Presentations (n = 29,698)			
30 day ED visit	2,580 (8.7)		
High volume	1,483 (7.8)	0.68 (0.51, 0.91) [0.01]	0.74 (0.52, 1.04) [0.08]
Low volume	1,097 (10.2)	Reference	Reference
30 day specialist visit	11,612 (39.1)		
High volume	8,769 (46.3)	2.56 (2.42, 2.71) [<0.001]	1.81 (1.68, 1.94) [<0.001]
Low volume	2,843 (26.4)	Reference	Reference
30 day death	255 (0.9)		
High volume	161 (0.9)	0.93 (0.26, 3.12) [0.91]	0.41 (0.07, 2.51) [0.33]
Low volume	94 (0.9)	Reference	Reference
90 day ED visit	3,910 (13.2)		
High volume	2,333(12.3)	0.75 (0.60, 0.94) [0.01]	0.81 (0.73, 0.90) [<0.001]
Low volume	1,577 (14.6)	Reference	Reference
90 day specialist visit	17,584 (59.2)		
High volume	12,810 (67.7)	2.85 (2.70, 3.02) [<0.001]	1.82 (1.76, 2.03) [<0.001]
Low volume	4,774 (44.3)	Reference	Reference
90 day death	613 (2.1)		
High volume	376 (2.0)	0.83 (0.38, 1.81) [0.63]	0.91 (0.32, 2.58) [0.86]
Low volume	237 (2.2)	Reference	Reference
Admitted Presentations with Hospitalization Record (n = 14,803) ‡			
30 day ED visit§	600 (4.1)		
High volume	212 (2.5)	0.39 (0.19, 0.81) [0.01]	0.37 (0.15, 0.87) [0.02]
Low volume	388 (6.1)	Reference	Reference
30 day death	780 (5.3)		
High volume	539 (6.4)	1.68 (0.78, 3.61) [0.19]	0.93 (0.38, 2.23) [0.86]
Low volume	241 (3.8)	Reference	Reference
90 day ED visit§	1,080 (7.3)		
High volume	429 (5.1)	0.46 (0.27, 0.78) [0.004]	0.48 (0.26, 0.89) [0.02]
Low volume	651 (10.2)	Reference	Reference
90 day death	1,364 (9.2)		
High volume	932 (11.1)	1.68 (0.96, 2.95) [0.07]	1.24 (0.63, 2.42) [0.53]
Low volume	432 (6.8)	Reference	Reference

*Adjusted for male, age, socio-economic proxy, rural, Charlson Comorbidity Index, prior comorbidities (diabetes, depression, hypertension, cancer, chronic pulmonary disease, myocardial infarction, heart failure, cardiac arrhythmias), fiscal year, day of week (Monday-Friday, Saturday-Sunday), time of day (0800–1359, 1600–2359, 0000–0759), triage level, cardioversion at index ED presentation, number of ED presentations for AFF in previous year, number of physician visits in previous year, and number of specialist visits in previous year.

‡Not all admissions could be linked with a hospitalization record.

§ Days after discharge from hospitalized index ED visit.

Discharged patients at HV EDs were also less likely to return to the ED for AFF at 90 days (aOR = 0.81, 95%CI 0.73, 0.90; p<0.001) than discharged patients at LV EDs. Patients discharged from HV EDs were more likely to have a specialist visit at 30 (aOR = 1.81, 95%CI 1.68, 1.94; p<0.001) and 90 (aOR = 1.82, 95%CI 1.76, 2.03; p<0.001) days than patients at LV EDs.

There was no evidence of statistically significant differences in deaths within 30 and 90 days for all presentations, the discharged presentation subgroup, or the admitted presentation subgroup (Table 2).

## Discussion

Using a comprehensive, reliable, population-based database for ED presentations, we identified familiarity based on the volume of patients seen annually as a factor to explain some of the practice variation observed in Alberta EDs with respect to the outcomes of patients with primary atrial fibrillation or flutter. For example, HV EDs admit AFF patients less frequently, their patients experience fewer relapses resulting in ED presentation, and their patients are seen by consultants more rapidly. Despite increased patient comorbidities in the HV sites, deaths were similar at 30 and 90 days.

Other investigators have found similar results for other cardiac conditions. For example, in patients presenting with heart failure during 1999–2009, medium and high volume EDs admitted patient less frequently and of patients treated and released, lower risks of the composite outcomes death/hospitalization/ED presentation were seen in the 30 and 90 days post-ED presentation than low volume EDs.[16] Deaths differed by volume in the HF study unlike our study of AFF. A study during 1999–2005 of newly diagnosed heart failure showed that urban patients were more likely to receive outpatient care and less likely to be hospitalized or present to the ED than rural patients.[17] Notably, 95% and 48% the presentations for AFF in our study were from urban patients in the high and LV EDs, respectively.

In Ontario during 2004–2010, patients presenting at higher volume EDs for chest pain were more likely to receive follow-up from any physician and from a cardiologist within 30 days of ED discharge.[18] We too saw an increase in specialist visit at 30 days at HV EDs, likely because they were all sites in urban or regional centres where access to specialists is more readily available. Other examples of the association with better outcome and HV ED presentation have been seen with out-of-hospital cardiac arrest.[19] Deaths in the ED or after admission are not common in patients presenting to the ED for AFF and we did not observe a relationship between ED volume and death.

What else could explain these results? In HV Canadian EDs, atrial fibrillation is treated more aggressively by emergency physicians that in other locations.[20] For example, although the method differs (i.e., chemical first, electrical first) within and among EDs, cardioversion is often attempted by emergency physicians when a clear history of onset < 48 hours is documented. In addition, electrical cardioversion often requires multiple physicians (e.g., procedural sedation, cardioversion) for safety, and HV EDs have more physician coverage to deal with overall volume and that may contribute to outcome. Most large volume (e.g., urban and regional) EDs are staffed by full-time emergency physicians whereas smaller volume EDs tend to be staffed by locums or primary care physicians with less training and/or experience. While Canadian atrial fibrillation guidelines exist,[2,3] they were not published at the time of the study and evaluation of their implementation has not been widely conducted or published. Finally, adoption of clinical practice guidelines, evidence-based medicine education, and research activities at HV sites likely influences the practice more than at LV sites.

Our study has several limitations. The databases accessed in this study are unable to capture all cases of acute AFF visits to the healthcare system, ‘‘true” incidence of disease, the severity of the AFF episode, granular details on treatments (e.g., rate control, investigations, ED treatments and out-patient use of anticoagulants) and all Aboriginals. In Canada, there are three Aboriginal peoples: First Nations, Métis, and Inuit, and these groups have been shown to be high users of the emergency health care system, especially for cardio-respiratory problems. The study only focused on follow-ups made to settings within the province; however, patients likely do not migrate rapidly over the short period following an ED presentation. It is not clear if all patients presenting to the ED with AFF need a cardiology or internal medicine follow-up visit and if the follow-up visits to cardiologists or internal medicine specialists were directly linked with the preceding ED presentation. As only administrative data were used to assess the effect of ED volume on outcomes, spurious findings may result.[21] We also do not know the experience and training of the physician caring for the patient and if a specialty consult occurred in the ED. Notwithstanding the above issues, this large study provides robust population-based health outcomes information for a single province.

## Conclusions

For Alberta EDs dealing with higher numbers of AFF patients on an annual basis, admission and AFF relapse to the ED are less common, referrals to specialists occur more frequently, and deaths occur similarly compared to EDs with lower annual numbers. The reasons for these differences deserve further attention.

## Supporting Information

S1 Table Characteristics by admitted and discharged disposition. (DOCX)
