# Supplementary material for: Volume Matters: Improved Outcomes for Patients Presenting to High-Volume Emergency Departments with Atrial Flutter and Fibrillation
Source: PLoS One. 2016 Nov 4;11(11):e0165894. doi: 10.1371/journal.pone.0165894 (PMC5096686; doi:10.1371/journal.pone.0165894)
Supplement: S1 Table — (DOCX) [file pone.0165894.s001.docx]

**S1 Table. Characteristics by admitted and discharged disposition.**

|  | **Admitted (n=15,506)** | | **Discharged (n=29,968)** | |
| --- | --- | --- | --- | --- |
|  | Low Volume | High Volume | Low Volume | High Volume |
| ED presentation for AFF, n | 6,777 | 8,729 | 10,770 | 18,928 |
| Female, n (%) | 3,385 (50.0) | 4,495 (51.5) | 4,839 (44.9) | 9,234 (48.8) |
| Age, mean (SD) | 71.7 (13.1) | 73.6 (12.5) | 69.4 (13.1) | 67.7 (13.6) |
| Socio-Economic Proxy, n (%) |  |  |  |  |
| Aged < 65 years |  |  |  |  |
| First Nations | 150 (2.2) | 42 (0.5) | 196 (1.8) | 146 (0.8) |
| Government Sponsored Programs | 302 (4.5) | 283 (3.2) | 505 (4.7) | 731 (3.9) |
| Human Services  Recipient | 109 (1.6) | 185 (2.2) | 157 (1.5) | 367 (1.9) |
| Other | 1,303 (19.2) | 1,449 (16.6) | 2,721 (25.3) | 6,053 (32.0) |
| Aged ≥65 years |  |  |  |  |
| First Nations | 144 (2.1) | 34 (0.4) | 120 (1.1) | 51 (0.3) |
| Non-First Nations | 4,769 (70.4) | 6,736 (77.2) | 7,071 (65.7) | 11,580 (61.2) |
| Residence, n (%) |  |  |  |  |
| Urban | 3,160 (46.6) | 8,101 (92.8) | 5,267 (48.9) | 18,063 (95.4) |
| Charlson Comorbidity Index, n  median (IQR) | 6,577  1 (0, 2) | 8,514  1 (0, 2) | 10,591  1 (0, 2) | 18,514  1 (0, 2) |
| Comorbidities, n (%) |  |  |  |  |
| Diabetes | 216 (3.2) | 378 (4.3) | 309 (2.9) | 495(2.6) |
| Depression | 571 (8.4) | 1,026 (11.8) | 934 (8.7) | 2,133 (11.3) |
| Hypertension | 3,044 (44.9) | 3,867 (44.3) | 4,929 (45.8) | 8,117 (42.9) |
| Dementia | 211 (3.1) | 395 (4.5) | 220 (2.0) | 365 (1.9) |
| Anemia | 103 (1.5) | 226 (2.6) | 167 (1.6) | 297 (1.6) |
| Renal failure | 166 (2.5) | 389 (4.5) | 234 (2.2) | 487 (2.6) |
| Cancer | 405 (6.0) | 773 (8.9) | 663 (6.2) | 1,145 (6.1) |
| Chronic pulmonary  disease | 1,111 (16.4) | 1,589 (18.2) | 1,581 (14.7) | 2,258 (11.9) |
| Myocardial infarction | 366 (5.4) | 535 (6.1) | 459 (4.3) | 963 (5.1) |
| Heart failure | 1,215 (17.9) | 1,783 (20.4) | 1,853 (17.2) | 2,307 (12.2) |
| Cardiac arrhythmias | 2,104 (31.1) | 2,538 (29.1) | 5,262 (48.9) | 7,164 (37.9) |
| Triage Level, n (%) |  |  |  |  |
| Resuscitation (1) | 48 (0.7) | 90 (1.0) | 28 (0.3) | 53 (0.3) |
| Emergency(2) | 959 (14.2) | 3,504 (40.1) | 1,042 (9.7) | 8,006 (42.3) |
| Urgent (3) | 3,920 (57.8) | 4,740 (54.3) | 5,069 (47.1) | 9,922 (52.4) |
| Semi-Urgent (4) | 692 (10.2) | 277 (3.2) | 1,681 (15.6) | 755 (4.0) |
| Non-Urgent (5) | 166 (2.5) | 17 (0.2) | 1,443 (13.4) | 105 (0.6) |
| Missing | 992 (14.6) | 101 (1.2) | 1,507 (14.0) | 87 (0.5) |
| Cardioversion, n (%) | 77 (1.1) | 517 (5.9) | 358 (3.3) | 3,398 (18.0) |
| Length of Stay in hours, n  median (IQR) | 6,323  2.1 (1.2, 3.8) | 8,078  7.0 (4.3, 11.2) | 9,759  2.1 (1.0, 4.2) | 18,073  4.8 (3.2, 7.5) |
| At least 1 ED presentation for AFF in previous 365 days, n (%) † | 976 (14.4) | 1,049 (12.0) | 2,910 (27.0) | 4,324 (22.8) |
| ED presentations for AFF in previous 365 days for those with previous ED visits, median (IQR) † | 1 (1, 2) | 1 (1, 2) | 1 (1, 2) | 1 (1, 2) |
| Physician office visits in previous 365 days, median (IQR) | 16 (8, 29) | 21 (10, 38) | 19 (10, 32) | 20 (10, 34) |
| Specialist office visits in previous 365 days, median (IQR) | 0 (0, 2) | 1 (0, 6) | 0 (0, 3) | 1 (0, 6) |

† 3,082 index ED visits for AFF were prior to April 1, 2000, and thus did not have the full 365 days prior of observation.
